# Editorial: Advances in the medical management of infantile hemangioma

**DOI:** 10.3389/fonc.2023.1229197

**Published:** 2023-06-16

**Authors:** Haiwei Wu, Yi Ji, Jiawei Zheng

**Affiliations:** ^1^ Department of Stomatology, Shandong Provincial Hospital Affiliated to Shandong First Medical University, Jinan, Shandong, China; ^2^ Division of Oncology, Department of Pediatric Surgery, West China Hospital of Sichuan University, Chengdu, Sichuan, China; ^3^ Department of Oromaxillofacial Head and Neck Oncology, Shanghai Ninth People’s Hospital, College of Stomatology, Shanghai Jiao Tong University School of Medicine, Shanghai, China

**Keywords:** infantile hemangioma (IH), medical management, propranolol, hypoxia, angiogenesis

Infantile hemangioma (IH) is a common vascular tumor that affects 4-5% of infants. If left untreated, IH can result in permanent changes, causing cosmetic dysfunction. Approximately 10% of IH cases are destructive and problematic, posing an increased risk of disfigurement and functional impairment. Consequently, early medical management is crucial to promote the rapid resolution of problematic IHs.

Although the beta-adrenergic receptor (β-AR) blocker propranolol (PRN) has been widely used as the first-line therapy for IH since 2008, concerns regarding its safety and resistance still persist. To address these issues, extensive clinical trials and basic studies are necessary to provide further support. Therefore, the objective of this Research Topic is to compile original research focusing on advancements in the medical treatment of IH. All these aspects have been comprehensively addressed in the present Research Topic, which has been successfully launched in *Frontiers in Oncology*.

Understanding the pathogenesis of hemangiomas is crucial for the development of new treatment approaches and minimizing treatment-related adverse reactions. Xu et al.‘s review explores the hypothesis of placental origin and the concept of a metastatic niche in IH. The placental origin hypothesis is supported by the expression of placental markers, such as laminin, merosin, and GLUT-1, in the blood vessels of hemangiomas. Additionally, GLUT-1-positive cells in IH possess stem cell characteristics and can differentiate into adipocytes, pericytes, or endothelial cells (ECs). This finding challenges the previous notion that ECs derived from hemangioma-derived stem cells (HemSCs) are the predominant tumoral cells in IH. Chen et al.‘s study identified CD146+ hemangioma mural cells (HemMCs) as the cell population exhibiting the greatest variability during the transition from the proliferation phase to the involution phase. Their research suggests that proangiogenic HemMCs could serve as potential targets for developing IH animal models and studying IH pathogenesis. In contrast to the placental origin hypothesis, a recent study by Moisan et al. demonstrated that hemangioma-derived endothelial cells (HemECs) lacked Aquaporin-1 (AQP1), whereas placental tissue-derived ECs exhibited AQP1 positivity (1). Additionally, the presence of a significant population of highly AQP1-positive telocytes in IH was proposed as a novel cell type within the tumor. IHs have been observed to occur as a secondary consequence of perinatal hypoxia. The hypoxic stress experienced during this period can trigger the overexpression of angiogenic factors, contributing to the development of IHs. As for detecting the hypoxic status in IH, the most regular methods are primarily to evaluate hypoxia-related biomarkers, rather than the hypoxic status itself. Wu et al. developed an ultrasensitive fluorescent chemosensor, HNT-NTR, to assess the hypoxic status of HemECs and investigate its potential for imaging purposes. Treatment of HemECs with low concentrations of PRN resulted in a significant reduction in real-time fluorescence signal, indicating the inhibition of hypoxia. This chemosensor provides an optical approach to visualize the therapeutic response of IH to PRN therapy, specifically targeting the hypoxic microenvironment.

Angiogenesis and vasculogenesis, mediated by ECs, play a crucial role in driving new vessel formation in IH. These processes necessitate a substantial energy supply to facilitate the proliferation and migration of ECs. Recently, the metabolic activity of ECs, particularly glycolytic metabolism, has emerged as a key driver of angiogenesis (2). Yang et al. discovered that PKM2, a critical glycolytic enzyme, promoted angiogenesis in HemECs, while suppression of PKM2 inhibited IH progression in a mouse model (3). Similarly, another study demonstrated that targeting FPFKB3 suppressed glycolysis and induced apoptosis in HemECs, effectively inhibiting angiogenesis (4). Furthermore, Li et al. demonstrated that the knockdown of TGFBI in HemECs resulted in the inhibition of glycolysis, and TGFBI played a role in enhancing angiogenesis and IH progression through its regulation of glycolysis in HemECs (5). Taken together, targeting glycolysis may represent a novel therapeutic approach for inhibiting the progression of IH and angiogenesis.

Accumulating evidence suggests that β-ARs play a role in hypoxia-induced angiogenesis, providing a new perspective on the pathogenesis of IH and potentially explaining the efficacy of β-blockers in IH treatment. Chen et al. reported three cases of abrupt deteriorations in IHs following the administration of β2-AR agonists. The enhanced vasodilation of IHs through the β2-AR-driven proangiogenic pathway resulted in increased size and redness, indicating the involvement of β-ARs in IH progression. However, another study demonstrated that the R (+) enantiomers of propranolol and atenolol inhibited vessel formation in HemSCs independently of β-ARs (6). Thus, the precise mechanisms underlying the effects of β-blockers and β-ARs on IH involution require further investigation.

Currently, there are various treatment options available for IH, including propranolol, atenolol, corticosteroids, and laser therapy. Oral PRN is commonly considered as a first-line systemic therapy for hemangiomas. However, it can lead to a range of complications. To mitigate the potential side effects of oral PRN, topical β-blockers have been employed as an alternative treatment approach for IH. Nonetheless, systemic absorption and associated adverse events remain a concern. Given the side effects associated with existing treatments, there is an urgent need to enhance both the therapeutic efficacy and safety of IH treatments. Wang et al. conducted a retrospective study involving 368 patients with IH who underwent low-dose lauromacrogol injection. The study reported excellent regression in 226 patients (61.4%) and good regression in 108 patients (29.4%). These findings demonstrate that this novel sclerotherapy approach accelerated the regression of IH without causing serious complications.

The advancements in nanotechnology have paved the way for nanomedicine, which holds the potential to enhance drug efficacy and minimize side effects by improving the pharmacokinetic and pharmacodynamic properties of conventional drugs. In this context, Wu et al. developed a novel drug delivery system for PRN by encapsulating it in mesoporous silica nanoparticles (MSN) (7). Their study revealed that this innovative nanodrug delivery system effectively suppressed the proliferation of HemSCs and inhibited the growth of IH in xenograft models. Additionally, the system demonstrated favorable attributes such as high therapeutic efficacy, reduced administration frequency, and low cytotoxicity, making it an attractive strategy for IH therapy. These findings highlight the potential application of nanomedicine-based therapeutic approaches in clinical practice in the foreseeable future.

## Conclusion

The primary objective of this Research Topic was to facilitate the progress of research and development in the field of medical management of IH by publishing high-quality research articles. The Research Topic featured five papers that yielded promising outcomes and introduced novel perspectives on the medical management of IH. We express our gratitude to all the authors for their valuable contributions and extend our appreciation to the referees for their meticulous review process.

## Author contributions

All authors have made a substantial, direct, and intellectual contribution to the work and approved it for publication.
